# Qualitative analysis of cognitive and social congruence in peer-assisted learning – The perspectives of medical students, student tutors and lecturers

**DOI:** 10.1080/10872981.2020.1801306

**Published:** 2020-08-03

**Authors:** Teresa Loda, Rebecca Erschens, Christoph Nikendei, Stephan Zipfel, Anne Herrmann-Werner

**Affiliations:** a Department of Psychosomatic Medicine and Psychotherapy, University Hospital Tuebingen, Tuebingen, Germany; b Centre for Psychosocial Medicine, Department of General Internal Medicine and Psychosomatics, University Hospital Heidelberg, Heidelberg, Germany; c Deanery of Students’ Affairs, University’s Faculty of Medicine, Tuebingen, Germany

**Keywords:** Cognitive congruence, social congruence, peer-assisted learning, medical students, student tutors, lecturers

## Abstract

**Background:**

The teaching of students by peers, so-called peer-assisted learning, is effective based on cognitive and social congruence among students and student tutors. This study aims to investigate cognitive and social congruence by analysing the perspectives of students, student tutors and lecturers in order to receive a better understanding of these concepts and to improve the teaching in tutorials as well as the relationship among students and student tutors.

**Methods:**

Cognitive and social congruence were assessed by conducting semi-structured interviews. An interview guide about teaching and relationship in tutorials was based on previous findings of Schmidt & Moust (1995) and Lockspeiser et al. (2008). The interviews were analysed inductively by using qualitative content analysis.

**Results:**

Twenty-nine participants were interviewed. The following categories were found for cognitive congruence: knowledge base, high expertise by student tutors, same language and effective knowledge transfer. Social congruence was represented by relaxed learning atmosphere, sharing social roles, empathic and supportive behaviour of student tutors, sharing experiences, understanding difficulties and enjoying tutorial.

**Conclusion:**

Cognitive and social congruence may be displayed on the practical behavioural level. Trust in student tutor could be found in both concepts on different levels. The qualitative results provide a deeper insight by indicating that the student tutors may play a ‘mediator’ role for the relationship between students and lectures.

## Introduction

Cognitive and social congruence are seen as key factors for an efficient peer-assisted learning (PAL) experience among students and student tutors [1–5]. PAL is defined as a well-established kind of teaching where students are taught by peers, so-called student tutors, who are mostly in higher years of study.

According to previous studies [1,5], cognitive congruence can be seen as a common and similar knowledge base between the student tutors and students. Further, using a familiar language contributes to cognitive congruence between the students and student tutors, as the student tutors explain difficult topics at an appropriate level [1,6,7]. Social congruence refers to the interpersonal level, as student tutors are interested in students’ problems and demands [8,9]. Furthermore, social congruence refers to the fact that students and student tutors share similar social roles [1,5]. Social congruence is also represented by high enjoyment and approachability [10,11].

The learning atmosphere is experienced as relaxed and pleasant by the students as well as student tutors when they are perceived as cognitively and socially congruent [12–14]. According to previous studies, students see their student tutors as cognitively and socially congruent when they share learning experiences or propose alternative solutions [1,5,15]. In turn, when tutors are perceived as cognitively and socially congruent students feel less anxious and dare to tell if they did not understand the topic [16–18].

Although PAL represents a valuable teaching tool in the general curricula, past studies postulated that it nevertheless remains under-researched [19–21]. The concept of cognitive and social congruence can be seen as a ‘black box’ in the PAL context. Many studies reported that PAL is effective due to cognitive and social congruence [22–25]. However, there is no empirical evidence regarding how cognitive and social congruence contribute to an effective PAL learning experience [24].

This study aims to investigate cognitive and social congruence with a focus on the student tutors’ and students’ relationship as well as the teaching in tutorials by conducting semi-structured interviews for thematic analysing. The perspectives of students as participants, of student tutors as teachers, and of lecturers as supervisors were considered to receive all perceptions of cognitive and social congruence in tutorials. To the best of our knowledge, this study presents the first investigation of cognitive and social congruence that contains the perspectives of all relevant persons involved in PAL (students, student tutors and lecturers).

## Methods

### Ethics

The study received ethical approval from the Ethics Committee of Tuebingen Medical Faculty (No. 129/2017BO2) in April 2017. All participants provided their written informed consent. As incentives for participation, books and vouchers were raffled among the participants.

### Design, participants and procedure

This study presents a cross-sectional method design with semi-structured interviews on cognitive and social congruence in student tutorials. Medical students, student tutors and lecturers from the Medical Faculty of Tuebingen were invited to participate. The medical students were from different years of study, ranging from the first until the final (for details, see Table 1). Student tutors came from various fields, such as medical history, anatomy, internal medicine, physiology, skills lab, and surgery. Medical students and tutors were recruited from different classes within their usual mandatory courses. All tutors were supposed to teach the course at least one time and had a didactic qualification. Lecturers were invited online. We were interested in the perspectives of all participants in student tutorials. Table 1 presents an overview of the participants. There no significant differences in age for students and tutors.

**Table 1.** Demographics of medical students, student tutors and lecturers.

	Students	Tutors	Lecturers
**N**	**13**	**10**	**6**
**Gender**	**m = 30.8%** **f = 69.2%**	**m = 10.0%** **f = 90.0%**	**m = 66.7%** **f = 33.3%**
**Age**	**M = 23.8** **SD = 2.7**	**M = 23.5** **SD = 2.2**	**M = 41.7** **SD = 9.5**

### Measurement

Demographic information such as gender, age, year of study and questions belonging to tutorials (e.g. subject of tutorial) were included in the interviews. The student tutors, additionally, were asked about their qualifications, their class rank and discipline as student tutors. Further, lecturers were also interviewed about demographic data such as age, gender, job, discipline and teaching experience.

#### Interview guide

A semi-structured interview guide was constructed for students, student tutors and lecturers based on previous evidence [1,9,26,27]. The interview guide was created by using the SPSS-method (*Sammeln* [collect], *Prüfen* [check], *Sortieren* [sort], *Subsumieren* [subsume]) developed by Helfferich [28]. First, questions were gathered by brainstorming. Second, they were checked for coverage of the topic. Third, questions were sorted by topic, and last, they were subsumed after Helfferich [28], meaning they were ranked and subordinated. The SPSS-method was used to structure our questions based on cognitive and social congruence and develop an interview guideline. For an overview of the interview guideline please see appendix.

The interviews started with open-ended questions, followed by more targeted ones Students and tutors answered the same questions, only from their respective perspectives. In order to investigate cognitive and social congruence with deeper insight on a behavioural level, they received questions about the teaching in student tutorials, the relation between students and student tutors in the tutorial, and general questions about the tutorial. Regarding the teaching, students and student tutors were asked about knowledge base, knowledge transfer, terminology, explanation of difficult topics and their experience. For the relationship between students and tutors, the questions were about communication, similarity, the student tutors’ interest in the students, their role model function, emotions and behaviour during lesson, and their motivation. General questions dealt with the learning environment in class, learning success, effectiveness and enjoyment of the tutorial. The lecturers had fewer but the same questions about knowledge transfer, students’ and tutors’ communication and similarities, learning environment, and effectiveness of the tutorial.

### Data processing

All data were pseudonymised. The interviews were recorded and transcribed verbatim. Recordings and transcripts were stored on a secure computer of the University Hospital with no connection to patient or Internet network.

### Data analysis

The interviews were analysed by using the qualitative content analysis by Mayring (2015). The aim of the analysis was to determine how the concept of cognitive and social congruence is represented in the student tutorials and reflected by the behaviour of students as participants and student tutors as teachers. The interviews were analysed inductively and iteratively by two independent raters (HL, TL), according to Mayring’s qualitative content analysis [29]. The raters followed the seven-step model of Mayring [29], including paraphrasing, reduction, summing up for general paraphrases, naming and describing of categories, adding examples, building hierarchy of categories, and recoding: At the beginning, the different analysis units get determined and the important text passages are paraphrased afterwards. Then, the desired level of abstraction gets determined and the paraphrases are generalised in regard of this level of abstraction . After that, the paraphrases are reduced by selecting the most important ones and by deleting paraphrases with the same meaning. In the next step, the paraphrases are reduced even further by bundling, constructing and integrating them at the desired level of abstraction. The new statements are compiled as a system of categories. In the last step, the summarised system of categories is reviewed by orientating on the source material.

The goal of the analysis and the theoretical background were defined according to the interview guide of the semi-structured interviews. Cognitive and social congruence were chosen as main category. Cognitive congruence referred to the knowledge transfer among student tutors and students. Social congruence referred to the relationship between student tutors and students. . In a second step, each of the raters started working through the material line by line and coded it by using MAXQDA Version 12.3.2. In order to obtain the same level of abstraction in building the categories, the raters revised them together and agreed on the final categories by paraphrasing representative examples and building a hierarchy of categories. Following this, both raters independently worked through the material again.

Audit trails were used to enhance trustworthiness and credibility of data analysis.

## Results

### Samples

N = 13 medical students, N = 10 student tutors and N = 6 lecturers were interviewed. The student tutors taught in average 2.85 years (SD = 1.34). The disciplines of the student tutors were anatomy, internal medicine, taking history, physiology and emergency medicine. The lecturers taught in average 7.1 years (SD = 5.1) and worked in the field of paediatrics, internal medicine, anatomy and surgery.

### Interviews

#### Results of cognitive congruence

**Figure 1.**
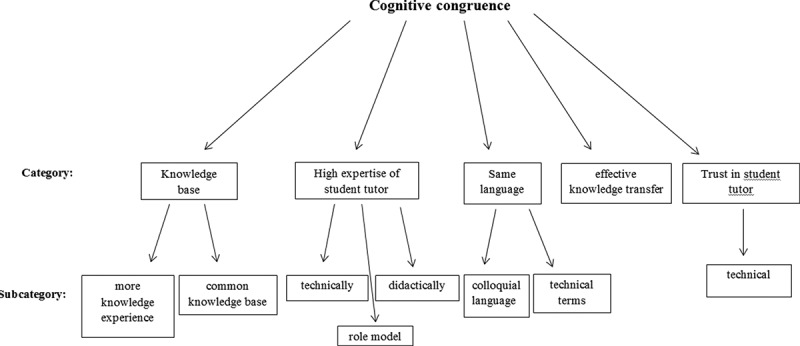
Categories and subcategories found for cognitive congruence based on qualitative analysis.

According to the analysis cognitive congruence was represented by the following categories: knowledge base, high expertise by student tutors, same language, effective knowledge transfer, and trust in student tutors. When suitable/possible, subcategories were rated from the answers of the participants for each category. Please see Figure 1 for an overview of the categories found for cognitive congruence.

#### Knowledge base

Knowledge base presents one relevant point in regard to cognitive congruence. Student tutors and students should have a common knowledge base, although the student tutors have advanced knowledge.

*‘At the beginning, the tutor had a higher level of education than us, but he tried to teach it to us. Of course, it was higher at the beginning, but it gradually became similar through the course of the tutorial.’* (Student 2)

*‘Comparing students and tutors, the difference is normally not that large, but tutors often have a special detailed knowledge.’* (Lecturer 5)

#### High expertise by student tutor

Further, student tutors are expected to have more expertise, both technically and didactically. At the professional level, they are also seen as role models.

*‘Student tutors take classes before they become student tutors. This is where they learn what they have to tell the students. If there are any remaining questions, you can always ask the lecturer. And I think that they are well-prepared within this training course.’* (Student 8)

*‘You don’t necessarily learn how to communicate in your studies. Nevertheless, tutors get better through the training, they are advanced in their studies.’* (Lecturer 6)

*‘Firstly, I found it impressive and exemplary that tutors are well-structured and know that much. I also see them as a role model, as they take so much time and really get involved, even though they have to learn a lot for their intermediate examination for medical students.’* (Student 9)

#### Same language

In the tutorial, tutors and students should share the same language. Many reported that technical terms as well as colloquial language are used.

*‘He* [student tutor] *used a simple language and no specialist language as a lecturer would use. In the chemistry tutorial, we called a cancer cell “Mr. Crabs” ‒ which a lecturer would never do. With funny memory hints.’* (Student 3)

*‘It is most important to watch your choice of words. When you start using too many specialist terms, you often get the problem that younger students don’t know them so far.’* (Student tutor 9)

#### Effective knowledge transfer

All three groups strengthen the effective knowledge transfer in the tutorial. Students receive a higher knowledge gain and tutors have progressed as students themselves in their curriculum.

*‘The information content of the tutorial was really good. I’m more of a practical person anyway. When I see something and work it out for myself, it’s easier for me to understand.’* (Student 10)

*‘They all* [students] *find it* [the tutorial] *very good, as they obtain a lot of knowledge in the tutorial and are able to a lot more things afterwards.’* (Student tutor 5)

*‘I think it [the tutorial] is definitely very efficient, as the students are closely accompanied* [by the student tutors], *much more than it would be possible with a medical colleague.’* (Lecturer 4).

#### Trust in student tutors

When trust in tutors is particularly technical, this might also represent a key factor for cognitive congruence.

*‘*

*I would definitely trust her* [student tutor] *for the content and technical knowledge. I also trust that when she says she doesn’t know it exactly and looks it up again. So I trust that she can estimate her professional competence of what she does know and is able to teach us, and what she does not.’* (Student 2)

#### Results of social congruence

**Figure 2.**
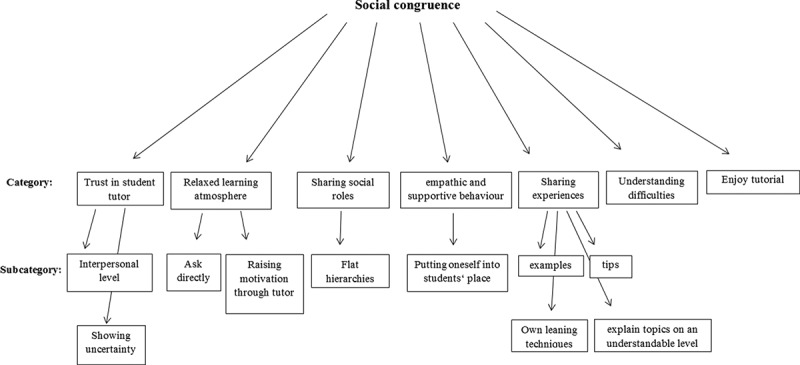
Categories and subcategories found for social congruence based on qualitative analysis.

Based on the evaluation of the interviews, the following categories of social congruence could be identified: trust in student tutors, relaxed learning atmosphere, asking questions directly, motivation, sharing social roles, empathic and supportive behaviour of the student tutors, sharing experiences and giving tips, understanding difficult topics, and enjoying the tutorial (see also Figure 2).

#### Trust in student tutors

In regard to social congruence, trust in student tutors was seen on an interpersonal level where students could show their uncertainty and contact tutors about problems.

*‘Here, again, it mostly applies to the younger students who recently moved away from home. Tutors like to ask.’*(Student 1)

*‘ … you can do things outside the tutorial, meaning not only professionally but also on a friendship basis.’* (Student tutor 1)

*‘ … it’s inevitable that they also ask about living situation, cooking recipes or whatever.’* (Lecturer 1)

#### Relaxed learning atmosphere

A relaxed learning atmosphere was shown as a relevant point for social congruence.

*‘ … the learning atmosphere is casual, but concentrated. It leaves room for entertaining moments. I think that’s really important for learning … You should be allowed to laugh, but you also need to convey what has to be achieved and which means you have for that.’* (Student 2)

*Motivated, concentrated, and relaxed. Such delightful learning.’* (Student tutor 2)

*‘Content should be conveyed with pleasure and fun. Especially, one [tutor] should pay attention to important details that need to be passed on to the students.’* (Lecturer 2)

#### Asking questions directly

Students should be allowed to ask questions directly.

‘*The interaction is more personal, and questions are asked more directly than if you would ask a lecturer.’* (Student 2)

*‘Everybody likes to ask, and one dares to ask questions.’* (Student tutor 3)

#### Motivation

Further, the student tutors could raise the students’ motivation for the tutorial.

*‘If you had a bad day, it could lift you up and motivate you.’* (Student 1)

*‘If the tutor was good, the motivation was always higher.’* (Student 3)

#### Sharing similar roles

All three groups reported that student tutors and students share similar roles, and in this context, the relevance of flat hierarchies.

*‘ … most of the time, these were people I could identify with more. These were people who rather like to have contact with others … That’s why I shared more with them than with others.’* (Student 4)

*‘*[We have the] *same job later, same student surroundings, same learning environment, problems and challenges.’* (Student tutor 4)

*‘A big advantage of our tutorial is that tutors and students are very close.’* (Lecturer 2)

#### Empathic and supportive behaviour of student tutor

Moreover, student tutors show empathic and supportive behaviour towards the students. They are able to put themselves into the students’ place.

*‘For lecturers, it has been a long time since they learned it. So they are not able to put themselves in the students’ place so easily. That’s why they formulate some things in a too-complicated or too-easy way. They cannot judge our knowledge level correctly. The student tutor is better at this.’* (Student 6)

*‘I show it once, and then they can practice themselves. I support them, explain something or answer questions.’* (Student tutor 5)

*‘They are empathetic and supportive. We request our tutors to walk around the rows and ask if there are problems or if there is a need for help.’* (Lecturer 5)

#### Sharing experiences and giving tips

In regard to social congruence, tutors share their experiences with the students by giving examples or tips. They also present their own learning techniques and explain topics on an understandable level.

*‘They give us advice based on their experience or what they felt to be important.’* (Student 6)

*‘I do share my experiences. Or what I also do is when I have friends from AGN* [Working Group Emergency] *who sometimes have been part of the rescue service for several years and have some practical advice that is not in the book, I invite them’* (Tutor 6)

#### Understanding difficult topics

Based on our findings, social congruence supports student tutors to understand the students’ difficulties with a topic as they are also students and thus, they mostly know the students’ difficulties.

*‘He could understand them well. I also told him when I had difficulties.’* (Student 3)

*‘We are better at understanding what difficulties might be. I was able to relate to the problems. At the beginning, when I became a tutor, this was also my problem to understand it well.’* (Student tutor 7)

*‘Indeed, you also think that tutors are closer to students’ problems or they are better at relating to them.’* (Lecturer 1)

#### Enjoying the tutorial

Finally, the tutorial should be fun so students and tutors might enjoy them.

*‘When you really learned something and didn’t have the feeling of wasting your time.’* (Student 9)

*‘I just really enjoy it because they all participate with such enthusiasm.’* (Student tutor 8)

*‘Student tutors giving us feedback say they have fun. Most of the time, they have already been student tutors in the dissection course before, and they enjoyed it, and then they also want to do a course in the clinical part.’* (Lecturer 1)

## Discussion

This study aimed to assess cognitive and social congruence with a focus on the behavioural level of students and student tutors by conducting interviews with all relevant persons involved, including students, student tutors and lecturers. Cognitive congruence was represented by the following categories: knowledge base, high expertise by student tutors, same language, effective knowledge transfer and trust in student tutors. For social congruence, we found trust in student tutors, relaxed learning atmosphere, asking questions directly, motivation, sharing social roles, empathic and supportive behaviour of the student tutors, sharing experiences and giving tips, understanding difficult topics, and enjoying the tutorial as associated categories

As many studies have already reported, cognitive congruence has been based on the same knowledge framework and similar language between the students and student tutors [1,2,5,25,27]. The results of this study further showed that the student tutors had an advanced knowledge that focuses on relevant details for the students’ learning [30]. Student tutors were expected to have more expertise, both technically and didactically, which might contribute to cognitive congruence between student tutors and students. Especially when regarding the professional level, they were seen as role models by the taught students. Schmidt [1] shows that expertise next to social congruence was a strong predictor for cognitive congruence. Other studies even postulated that students did not miss the expertise of the student tutors, as it was compensated by cognitive congruence [5,31,32]. Besides, all three groups including students, student tutors and lecturers strengthened the effective knowledge transfer in the tutorial. This knowledge transfer might be associated with cognitive congruence, as the students were closely accompanied by the student tutors when practicing in the tutorial. Many studies reported that PAL is effective due to cognitive congruence [5,19,33,34]. Consequently, the present study assumes that the effective knowledge transfer that contributed to cognitive congruence might be one relevant factor for effective learning success in PAL. Regarding cognitive congruence, students reported that they trust their student tutors on the content and professional level. They had trust in their knowledge and skills and feel comfortable when the student tutors do not know something and looked at it for the next class.

Trust in tutors was reported as a key element for social congruence. In this context, trust in student tutors was regarded on an interpersonal level, where students could show their uncertainty and contact student tutors regarding private problems they had. Previous studies also show this kind of interpersonal trust is built by establishing social congruence [35,36]. In contrast to previous studies, a relaxed learning atmosphere was reported as a relevant point for social congruence and not for cognitive congruence [13,14]. De Menezes (2016) argued that cognitive congruence promotes a less-stressful learning environment because students are taught at an appropriate level of difficulty and feel less intimidated [13]. Hence, in this study, a relaxed learning atmosphere was associated with entertaining moments. Even one lecturer said that the tutorial should be conveyed with pleasure and fun (Lecturer 2). As expected, student tutors were perceived as socially congruent when students felt free to ask questions directly [16,37,38]. Further, student tutors could increase the students’ motivation by showing social congruence, which was also reported by Khaw [39]. All three groups reported that student and student tutors shared similar social roles, as they were able to identify with each other, which might have an impact on social congruence [1,25,27]. In accordance with previous study, student tutors were perceived as socially congruent when they behaved empathically and supportively [1,5,9]. Thus, they are able to put themselves into the students’ place. These results, further, imply that students might be less afraid in student tutorials as they dare to ask questions or to admit that they did not understand a difficult topic [5]. When showing social congruence, student tutors also shared their experiences with the students by giving tips or presenting their own learning techniques. Student tutors might also understand the difficulties of students due to social congruence [1,5,9]. Finally, social congruence contributed to the fact that students as well as student tutors had fun and enjoyed the tutorial.

### Role of student tutors in medical training

Student tutorial presents a relevant key element in medical training [25,26,40]. Here, knowledge is effectively imparted to the students by student tutors [41,42]. Tutorials, however, are not only effective due to the knowledge transfer. The social role of the tutors is also relevant, as student tutors deal with students as individuals during the knowledge transfer [24,37,43]. Therefore, empathy and individual support are relevant factors with regard to efficiency [9,16,17]. However, this study showed that these factors might be underestimated by students and tutors, because they perceived them unconsciously. The lecturers were more aware of these factors and should transfer them to the student tutors during the training in order to foster cognitive and social congruence. The results of this study further showed that the student tutors were much closer to the students so the students were less afraid of the student tutors as teachers [44]. This raises the question of whether student tutors act as mediator between the students and lecturers.

From a learning theory point of view student tutors might contribute to an higher knowledge gain but it highly depends on students’ motivation and self-confidence [33]. Student tutors could enhance the students’ motivation and support them in their learning. Though, students themselves are responsible for their knowledge gain.

### Implications for student tutor training

Based on the presented results, relevant and practical recommendations for action can be derived for future student tutor training. Student tutors should continue to have the same knowledge base and language. However, student tutors should be aware of the fact that students expect them to have special detail knowledge, and they have high confidence in their knowledge and skills from a professional perspective. On the other side, students trusted their student tutors when having private difficulties. The high trust of students in their student tutors on a professional and interpersonal level should also be focused on in the training of student tutors. Further, student tutors should learn how to create a relaxed learning atmosphere and to behave empathically and supportively. They were allowed to give advice and share their learning experience with the students. Sharing social roles with students should be a relevant part of the training, as students and student tutors should be on par with each other. Finally, one important point should be that all participants should have fun and enjoy the tutorial.

#### Limitations

Generalization of the results is limited, as this study was only representative of the medical students and lectures of the Medical Faculty of Tuebingen. Future studies should conduct semi-structured interviews based on the reported interview guide at other locations. The results are also limited by restricting the content to cognitive and social congruence and not regarding tutorials in general. We focused on cognitive and social congruence in order to enhance the effectiveness like knowledge transfer in tutorials and the relationship between students and student tutors. Further interesting topics like having trust in tutors were found during the analysis. These results should be investigated more closely in future PAL research. Here, future studies could also focus on the role of student tutors as they might influence the relationship between students and lectures. Moreover, there might be gender differences in the results as more students and tutors were female while lecturers were mostly male. This could impact on interactive behaviour in tutorials and should be investigated in further studies.

## Conclusion

This study assessed cognitive and social congruence on the behavioural level with a focus on students’, student tutors’ and lecturers’ perspectives. Cognitive congruence was represented by the same knowledge base and familiar language as well as an efficient knowledge transfer with detailed knowledge of the student tutors. Social congruence was shown by a relaxed learning atmosphere, an enjoyable tutorial, empathic and supportive behaviour of the student tutors, and sharing learning experiences. Further, student tutors were perceived as socially congruent, as they share similar roles and understand the students’ difficulties. Trust in students played an important role in cognitive as well as in social congruence, dependent on the professional or interpersonal level.

## Data Availability

Data are available from the corresponding editor upon reasonable request.

## Appendix Interview guiding questionnaire measuring cognitive and social congruency

Interview guiding questionnaire (student’s version)

Questions concerning cognitive congruence

1. How did your tutor design the tutorial?

(1.1) Overall, how were the tutor’s lessons compared to the docent’s lessons?
(1.2) How effectively did the tutor use the tutorial itself to convey knowledge?
(1.3) What kind of language/terminology did you and your tutor use?
(1.4) How would you rate the expertise of your tutor?

(2) Which ways of communication did your tutor use in the tutorial?

(2.1) How did your tutor explain/teach you difficult topics?
(2.2) To what extent was your tutor capable of understanding students’ problems?

(3) Which topics did you feel were particularly difficult and challenging?

Questions concerning social congruence:

1. How was your relationship to the other students?

(1.1) What do you and your tutor have in common?
(1.2) How interested do you think the tutor is in his/her students?

(2) I have already asked you about the ways of communication and to what extent your tutor gave you helpful advice or alternative solutions. Now I would like to know what the communication/interaction between you and your tutor was like on an emotional level.

(2.1) Describe your behavior and emotions during the lessons: How did you feel during the lessons?

(3) How would you evaluate your tutor? Describe the tutor’s behavior and emotions towards you as students during the lessons.

(3.1) To what extent did your tutor seem sympathetic and supportive towards the students?
(3.2) How did your tutor express praise and criticism?

(4) Allover, how motivated were you regarding the tutorials?

(4.1) Did your tutor affect your motivation

(5) Did you enjoy the tutorials?

Interview guide (lecturers’ version)

Questions about teaching the tutorial and about the relationship between tutor and students

(1) How would you describe the communication between the students and the tutor?

(1.1) How well did tutors and students get along?

(2) What do you think students and tutors have in common?

(2.1) Regarding the knowledge base?
(2.2) Regarding language/terminology?
(2.3) On an interpersonal level?

(3) How would you describe the learning atmosphere in tutorials?
(4) How would you describe the effectiveness atmosphere in tutorials?
(5) How would you describe your interaction with the tutors?
